# A psychosocial goal-setting and manualised support intervention for independence in dementia (NIDUS-Family) versus goal setting and routine care: a single-masked, phase 3, superiority, randomised controlled trial

**DOI:** 10.1016/S2666-7568(23)00262-3

**Published:** 2024-02

**Authors:** Claudia Cooper, Victoria Vickerstaff, Julie Barber, Rosemary Phillips, Margaret Ogden, Kate Walters, Iain Lang, Penny Rapaport, Vasiliki Orgeta, Kenneth Rockwood, Sara Banks, Marina Palomo, Laurie T Butler, Kathyrn Lord, Gill Livingston, Sube Banerjee, Jill Manthorpe, Briony Dow, Juanita Hoe, Rachael Hunter, Quincy Samus, Jessica Budgett

**Affiliations:** a Centre for Psychiatry and Mental Health, Wolfson Institute of Population Health, Queen Mary University London, London, UK; b Research Department of Primary Care and Population Health, University College London, London, UK; c Department of Statistical Science, University College London, London, UK; d Division of Psychiatry, University College London, London, UK; e Research Network Volunteer, Alzheimer's Society, London, UK; f St Luke's Campus, University of Exeter, Exeter, UK; g Division of Geriatric Medicine, Dalhousie University, Halifax, NS, Canada; h Faculty of Science and Engineering, Anglia Ruskin University, Chelmsford, UK; i Centre for Applied Dementia Studies, University of Bradford, Bradford, UK; j Faculty of Medicine and Health Sciences, University of Nottingham, Nottingham, UK; k The Policy Institute at King's, King's College London, London, UK; l National Ageing Research Institute, Melbourne, VIC, Australia; m Geller Institute of Ageing and Memory, School of Biomedical Sciences, University of West London, London, UK; n Department of Psychiatry and Behavioral Sciences, Johns Hopkins University, Baltimore, MD, USA

## Abstract

**Background:**

Although national guidelines recommend that everyone with dementia receives personalised post-diagnostic support, few do. Unlike previous interventions that improved personalised outcomes in people with dementia, the NIDUS-Family intervention is fully manualised and deliverable by trained and supervised, non-clinical facilitators. We aimed to investigate the effectiveness of home-based goal setting plus NIDUS-Family in supporting the attainment of personalised goals set by people with dementia and their carers.

**Methods:**

We did a two-arm, single-masked, multi-site, randomised, clinical trial recruiting patient–carer dyads from community settings. We randomly assigned dyads to either home-based goal setting plus NIDUS-Family or goal setting and routine care (control). Randomisation was blocked and stratified by site (2:1; intervention to control), with allocations assigned via a remote web-based system. NIDUS-Family is tailored to goals set by dyads by selecting modules involving behavioural interventions, carer support, psychoeducation, communication and coping skills, enablement, and environmental adaptations. The intervention involved six to eight video-call or telephone sessions (or in person when COVID-19-related restrictions allowed) over 6 months, then telephone follow-ups every 2–3 months for 6 months. The primary outcome was carer-rated goal attainment scaling (GAS) score at 12 months. Analyses were done by intention to treat. This trial is registered with the ISRCTN registry, ISRCTN11425138.

**Findings:**

Between April 30, 2020, and May 9, 2021, we assessed 1083 potential dyads for eligibility, 781 (72·1%) of whom were excluded. Of 302 eligible dyads, we randomly assigned 98 (32·4%) to the control group and 204 (67·5%) to the intervention group. The mean age of participants with dementia was 79·9 years (SD 8·2), 169 (56%) were women, and 133 (44%) were men. 247 (82%) dyads completed the primary outcome, which favoured the intervention (mean GAS score at 12 months 58·7 [SD 13·0; n=163] *vs* 49·0 [14·1; n=84]; adjusted difference in means 10·23 [95% CI 5·75–14·71]; p<0·001). 31 (15·2%) participants in the intervention group and 14 (14·3%) in the control group experienced serious adverse events.

**Interpretation:**

To our knowledge, NIDUS-Family is the first readily scalable intervention for people with dementia and their family carers that improves attainment of personalised goals. We therefore recommend that it be implemented in health and care services.

**Funding:**

UK Alzheimer's Society.

## Introduction

Around 885 000 people in the UK have dementia. This figure is projected to double within 25 years and health and social care costs are expected to triple to £80·1 billion by 2040. Most people living with dementia want to remain at home.^1^ 61% of those aged over 65 years with dementia in the UK live in their own homes, rather than in care homes. However, unmet needs, poor self-care, home safety risks, and burden reported by family, friends, unpaid carers, and caregivers (henceforth described as carers) are common reasons necessitating a move to a care home.^2^

The National Health Service (NHS) England's Well Pathway for Dementia and other initiatives stress the importance of promoting independence for people with dementia, which means living with good quality of life, choice, autonomy, dignity, and as independently as possible. The UK National Institute for Health and Care Excellence (NICE) dementia guidelines recommend offering people with dementia “psychosocial and environmental interventions to reduce distress” and personalised strategies for behavioural and sleep disturbance, and providing support for carers.^3^ Such interventions might reduce non-cognitive dementia symptoms and behaviours that challenge, with best available evidence showing efficacy of cognitive behavioural therapies,^4^ but which are seldom offered in practice.

**Research in context**

**Evidence before this study**

We searched PubMed, Embase (Ovid), and PsychINFO (Ovid) from Jan 1, 2012, to May 16, 2018, using the terms (dementia) OR (Alzheimer*), combined with intervention terms (non-pharmacologic*) OR (nonpharmacologic*) OR (psychotherapy) OR (rehabilitation) OR (“physical therapy”) OR (“goal attainment”) for studies examining the effectiveness of non-pharmacological interventions in reducing functional decline or improving individualised global outcomes (goal attainment) in people living in their own homes with dementia. We excluded studies that investigated nutritional interventions or interventions targeting caregiver-focused outcomes only that did not include components targeting care recipient-focused outcomes, measures of general rather than specific physical functioning (eg, mobility or balance), and studies in which either the intervention or control group had fewer than 15 participants to minimise bias. We identified 13 randomised controlled trials (RCTs) judged to have a low risk of bias, of which three described interventions that were associated with improved functioning. These were a 1-year, in-home, physical exercise programme delivered by physiotherapists; an in-home activities of daily living training and environmental strategy intervention (tailored activity programme [TAP]) delivered by occupational therapists; and 3 months of cognitive rehabilitation and activities of daily living training by psychologists. All were delivered individually and tailored to client needs. We updated the search on Aug 25, 2023, identifying two further RCTs of interventions, which included a relevant outcome. One compared ten cognitive rehabilitation sessions over 3 months, followed by four maintenance sessions over 6 months, delivered in participants’ homes by occupational therapists or nurses, to the treatment-as-usual control. The intervention was associated with significantly improved participant-rated goal attainment at 3 months (primary outcome) that was sustained at 9 months. A second RCT investigated TAP among 250 dyads of patients with clinically significant agitation or aggression and their carers. TAP did not reduce aggression, the primary outcome, compared with attention control, but the intervention was associated with less assistance with instrumental activities of daily living and activities of daily living (secondary outcomes) at 6 months.

**Added value of this study**

The NIDUS-Family intervention was effective in increasing attainment of dyads’ goals. It is, to our knowledge, the first intervention to improve goal attainment in people living with dementia that is potentially scalable, can be delivered by people without clinical training, and can be delivered remotely.

**Implications of all the available evidence**

The few non-pharmacological interventions done in people living with dementia showing effectiveness in RCTs were planned around personal goals and NIDUS-Family is, to our knowledge, the first evidence-based, manualised intervention that can enable care focused on personal goal attainment to be widely implemented. We recommend that post-diagnostic services routinely provide goal-focused, structured, manualised support to all people diagnosed with dementia who have a regular carer.

In systematic reviews,^5^, ^6^ we found no manualised interventions showing effectiveness in improving attainment of personalised goals or improving functioning in people with dementia living in their own homes. One randomised controlled trial (RCT)^7^ included in this review showed that an in-home, tailored, physical exercise programme delivered by physiotherapists improved physical functioning over 1 year. An RCT published subsequently, however, showed that an intensive, physiotherapist-delivered training programme for exercise and functional activity did not improve activities of daily living, physical activity, or quality of life, despite good uptake.^8^ Another RCT found that an individual, goal-oriented, cognitive rehabilitation by nurses and occupational therapists improved self-rated goal attainment in people with mild to moderate dementia.^9^

Psychosocial interventions that are fully structured and manual-based, allowing for consistent delivery (ie, standardised delivery), can be facilitated by trained, supervised staff without clinical qualifications, increasing the workforce and therefore increasing access to evidence-based dementia care. Examples include cognitive stimulation therapy, which improves cognition,^10^ and the Strategies for Relatives (START) intervention, which reduces psychological morbidity of carers.^11^

Standardised therapies might at first seem discordant with providing interventions that are personalised, which recognises that care is most effective when individually tailored. Goal setting is a prerequisite for personalising care. We co-designed, with patient and public involvement (involvement of lay representatives in the research, including people with personal or care experience of dementia), the New Intervention for Independence in Dementia Study–Family (NIDUS-Family) psychosocial support intervention to be fully manualised, modular so it can be tailored to individual goals, and delivered by facilitators without formal clinical training so that it is scalable for widespread delivery.

The NIDUS-Family intervention logic model^12^ and pilot study^13^ are reported elsewhere. We aimed to test our hypothesis that goal setting, NIDUS-Family, and routine care would be more effective in terms of the primary outcome of carer-rated goal-attainment scaling (GAS), compared with the control condition (goal setting and routine care), after 12 months.

## Methods

### Study design and participants

NIDUS-Family was a two-armed, parallel-group, single-masked, multi-site, superiority RCT. The protocol has been published previously.^14^

We recruited potential participants via professionals working in the community, NHS memory clinics, mental health services for older adults, and general practitioner practices (in London, Bradford, Leeds, Hull, Oxfordshire, Buckinghamshire, Kent, and Surrey) and via the recruitment database Join Dementia Research, X (formerly Twitter), and newspaper advertisements. We included dyads of people with dementia and their carers, in which the person with dementia had a documented dementia diagnosis of any type and any severity and lived in their own home and in which the carer had at least weekly face-to-face or telephone contact with the patient and spoke English. We excluded dyads if either member was enrolled in another research study, the person with dementia was in the last 6 months of life, or the carer lacked capacity to consent or could not identify at least three eligible GAS goals. Sex was self-reported.

Camden and King's Cross Research Ethics Committee (19/LO/1667) approved the study on Jan 7, 2020. Two substantial amendments to the protocol (approved on April 7, 2020, and Sept 19, 2022) were made. The first, in response to the start of the COVID-19 pandemic before study commencement, allowed for informed consent, outcome measures, and intervention delivery to be done remotely via telephone or video call. The second added procedures for a process evaluation^12^ and pre-implementation study. Additional 18-month and 24-month follow-ups (ongoing) were also added.

### Randomisation and masking

Allocation was assigned through a remote web-based system provided by PRIMENT Clinical Trials Unit (CTU; University College London). Individual randomisation was blocked and stratified by site using a 2:1 allocation ratio (intervention:control). Randomisation status was concealed from researchers completing outcome measures with carers, and researchers were asked to guess allocation after completing outcomes to assess masking success. We could not mask participants or facilitators.

### Procedures

Trained researchers obtained verbally recorded or written informed consent from all participating carers and people with dementia with capacity to consent; carers of people who lacked capacity completed a consultee declaration form. Because of pandemic-related restrictions imposed before study commencement, consent was obtained and assessments were done via telephone or video call, depending on the individual's preference; from April, 2021, when restrictions were lifted, we also offered in-person assessments. Data were collected at baseline, 6 months, and 12 months post-randomisation. Participants were offered a £20 voucher per assessment. Intervention sessions were audio recorded if participants agreed. All participants received routine care and completed goal setting before randomisation.

NIDUS-Family modules drew on behavioural management techniques (DICE approach^15^), enablement strategies, communication strategies, carer support strategies, and psychoeducation strategies, with material developed from existing interventions^11^, ^16^, ^17^, ^18^ and created by the co-production group. We originally designed NIDUS-Family for face-to-face delivery but, when COVID-19 restrictions were imposed, our co-production group adapted it for remote delivery.

NIDUS-Family was delivered by university-employed facilitators, without previous clinical training or clinical qualifications. Initial training for facilitators was manualised and comprised ten 1-h sessions, led by team members including a psychiatrist (CC), clinical psychologists (MP, PR, and SBank), and trial manager (JBu), with Alzheimer's Society research network volunteers (MO and RP). Training focused on introducing the programme clinical skills (two sessions), GAS (two sessions), and specific modules discussing delivery strategies (five sessions). Facilitators recorded completion of specified self-directed learning activities in their manual. These activities involved role-playing modules and goal-setting interviews, with some of these activities observed by the NIDUS team. In total, training took around 9 days. Facilitator competency was assessed (by SBank, MP, PR, or CC) via role-plays before intervention delivery. Facilitators attended group supervision with a clinical psychologist every 2 weeks.

Our trial was delivered over 12 months, with six to eight manualised sessions in the first 6 months, by video call or telephone (in person when COVID-19 restrictions permitted). In session one, the facilitator explored the person with dementia's life story and, with the participant, mapped their baseline goals to a module menu on the basis of their priorities and concerns. Facilitators and participants explored support networks and identified gaps, with facilitators signposting to existing resources and services. The modules included information and strategies addressing: (1) accepting care, arranging and planning for the future; (2) communicating with people living with dementia, family, and professionals; (3) managing behaviours that challenge (including agitation, aggression, and other distress behaviours); (4) managing physical health conditions; (5) exercise, activity, and mobility; (6) managing low mood, anxiety, and apathy; (7) carer wellbeing and support; (8) environmental and telecare adaptations to address safety concerns and supporting functioning at home; (9) relaxation; and (10) sleep, diet, and healthy routines.

Each selected module was completed over one to three sessions; dyads completed two to four modules in total. In the final sixth, seventh, or eighth session (depending on the preference of the dyad), the facilitators and participants reviewed helpful strategies, including those that have worked well previously and previous and new strategies developed during the intervention to formulate an action plan. Sessions included carers and people with dementia together, or just the carer. The most appropriate arrangement was agreed with dyads (depending on the session focus and circumstances). These manualised sessions were followed by 30-min catch-up telephone or video calls at 2–3-month intervals (at the preference of the participating dyad), taking place 6–12 months from baseline, to review progress towards goals**,** implementation of action plans, and to troubleshoot difficulties following a standard guide.

For goal setting, trained researchers worked with carers and people living with dementia to set three to five SMART (specific, measurable, achievable, realistic, and time-bound) goals across domains, including cognition, instrumental activities of daily living and self-care, mood, behaviour, and mobility. Any goal that carers considered important for the person with dementia to live well or independently at home over the next year within the intervention remit was permissible. Goals could be set around carer wellbeing or support when this outcome affected the functioning or wellbeing of the person with dementia, but at least one goal needed to relate directly to the person with dementia.

Participants in the control condition received usual routine care and completed goal setting.

### Outcomes

The primary outcome was carer-rated GAS score at 12-month follow-up. GAS is an individualised global outcome that measures goal attainment.^19^ GAS is valid, reliable, and responsive to change in people with dementia living at home.^20^

At follow-up, carers assessed goal attainment on a 5-point scale, ranging from “much worse” (–2) to “much better” (+2) than expected, with the expectation set by carers along with the facilitator at baseline. The baseline goal attainment was scored as zero. Because people had different goals and numbers of goals, we used the following summary formula to standardise degree of goal attainment:

$$T=50+\frac{10\sum x_{i}}{\surd \left(1-\rho \right)n+\rho n^{2}}$$

where *x*_i_ is the degree of goal attainment (–2 to +2), ρ is the expected overall intercorrelation between goal areas (typically 0·3), and *n* is the number of goals.

T=50 can be interpreted as meeting baseline expectations (no change), T<50 as not meeting, and T>50 as exceeding baseline expectations. Possible scores ranged from 0 to 100. Two authors (CC or JBu) reviewed all goals (for relevance to the intervention and equidistance between outcome scale descriptors) before their confirmation with the dyad, and KR reviewed a proportion of goals, in line with best GAS practice.^21^

For people with dementia who died in the preceding 6 months, GAS was rated as –2 at the next follow-up if death was unexpected and, if death was expected, the carer was asked to rate GAS on the basis of the 4 weeks before death.

Prespecified secondary outcomes were measured at 6 and 12 months. Secondary outcomes were (1) 6-month carer-rated GAS and researcher-rated GAS at 6 months and 12 months (with this rating based on their discussions with the dyad while completing other outcome assessments); (2) carer-rated performance of basic and instrumental activities of daily living and leisure activities in the 2 weeks before assessments, measured by the Disability Assessment for Dementia (DAD) scale^22^—the DAD scale records the number of activities the individual has had an opportunity to attempt that were performed without any assistance or prompting, and excludes activities that participants either did not have the opportunity to perform, or did not perform before a dementia diagnosis; (3) quality of life of the person living with dementia, measured by the carer-rated Dementia Quality of Life (DEMQOL) proxy scale,^23^ and, if they were able to, by the person with dementia using DEMQOL; (4) neuropsychiatric symptoms, using the Neuropsychiatric Inventory;^24^ (5) apathy, using the brief Dimensional Apathy Scale,^25^ which provides executive, emotional, and initiation apathy subscales (caseness on each scale was predefined; appendix p 6); (6) carer anxiety and depression, using the Hospital Anxiety and Depression Scale (HADS);^26^ (7) potentially abusive behaviours of carers, using the Modified Conflict Tactics Scale^27^ (caseness was predefined as a score of ≥2); (8) service use and care costs, using the Client Service Receipt Inventory; (9) time spent living at home during the study, up to the point of, if they occur, hospitalisation without return home, move to a care home, or death; and (10) carer quality of life, measured by the CarerQol instrument.^28^ Duration of time living at home, carer quality of life, and Client Service Receipt Inventory are part of our health economic analysis and will be reported separately.

Adverse events occurring within a year of randomisation, deaths, and care home moves (temporary or permanent) were reported by allocation group. The number of intervention sessions attended was reported. We purposively selected a fifth of recorded sessions across all facilitators, modules, and participant types (carer only or dyad), for which researchers completed fidelity checklists. Developed by the study team, these checklists evaluated whether facilitators kept participants engaged, focused on material, and to time, on five-item Likert scales.

### Statistical analysis

Our a priori sample size calculation indicated that 297 (198 in the intervention group and 99 in the control group) participants were required to detect a moderate effect size of 0·5 for the primary outcome comparison between intervention and control groups at a 5% significance level (two-tailed) with 90% power. The calculation included inflation for facilitator clustering in the intervention group (intracluster correlation coefficient 0·05; average cluster size 20) and 15% loss to follow-up.^26^

The primary outcome was summarised and compared between allocation groups using a three-level mixed effects model allowing for facilitator clustering in the intervention group and a random effect for study site. The control group was treated as a single cluster. This model estimated the treatment effect of adjusted mean difference in GAS score. All analyses were based on the intention-to-treat principle. Analyses of continuous secondary outcome scores took a similar approach to primary analyses, using linear models, additionally adjusting for the associated baseline measurement. When residuals for linear regressions performed for variables were not normally distributed, we used ordered logistic regression.

In sensitivity analyses, primary and secondary outcome models were refitted, adjusting for baseline predictors of missing data, which were identified by comparing characteristics of participants with and without missing outcomes using logistic regression models. For the primary outcome, we also imputed missing values, separately by randomised group and including baseline demographics, 6-month GAS score, site, and facilitator in the models. Finally, for GAS scores at 12 months, we did an analysis in which missing values for those who died or were known to have moved to a care home were classified as missing not at random. For these participants, we initially assumed missing follow-up GAS scores for each goal took a value of –2. We refitted the model using these imputed scores, then repeated it assuming goal scores of –1 for these participants.

We did an analysis adjusting for prespecified baseline factors: time of randomisation in months since the first participant was randomly assigned, level of overall functioning (DAD score), and carer stress (HADS score). We also fitted a model adjusting for observed imbalances in other baseline characteristics. For primary and secondary outcomes, models were refitted with an extra level to accommodate repeated measurements at 6 and 12 months and including fixed terms for timepoint and treatment group by timepoint interactions.

We investigated whether the primary outcome treatment effect differed by participant subgroups, defined by whether the person living with dementia had capacity to consent (a proxy for dementia severity), whether they indicated milder symptoms, and whether dyads were living together. For this analysis, we added subgroup by treatment interaction terms to the primary model. Given concerns about the impact of COVID-19 lockdowns on goal setting, attainment, and the intervention effect, we refitted the primary analysis model to include an interaction term that allowed the treatment effect to vary by calendar date of participant recruitment, relative to randomisation of the first trial participant. We also examined differences in the intervention effect depending on delivery mode by refitting the primary analysis model with treatment represented by three groups: video or face-to-face, telephone, and control condition. We estimated intracluster correlation coefficients to quantify the amount of variability in GAS outcomes that was due to facilitator clustering. We calculated the intracluster correlation coefficient in two ways: within the intervention group (considering variability of GAS outcomes only in that group) and considering variability and clustering across both groups. The full statistical analysis plan is provided in the appendix (p 1).

### Role of the funding source

The funder of the study had no role in study design, data collection, data analysis, data interpretation, or writing of the report.

## Results

**Figure 1:**
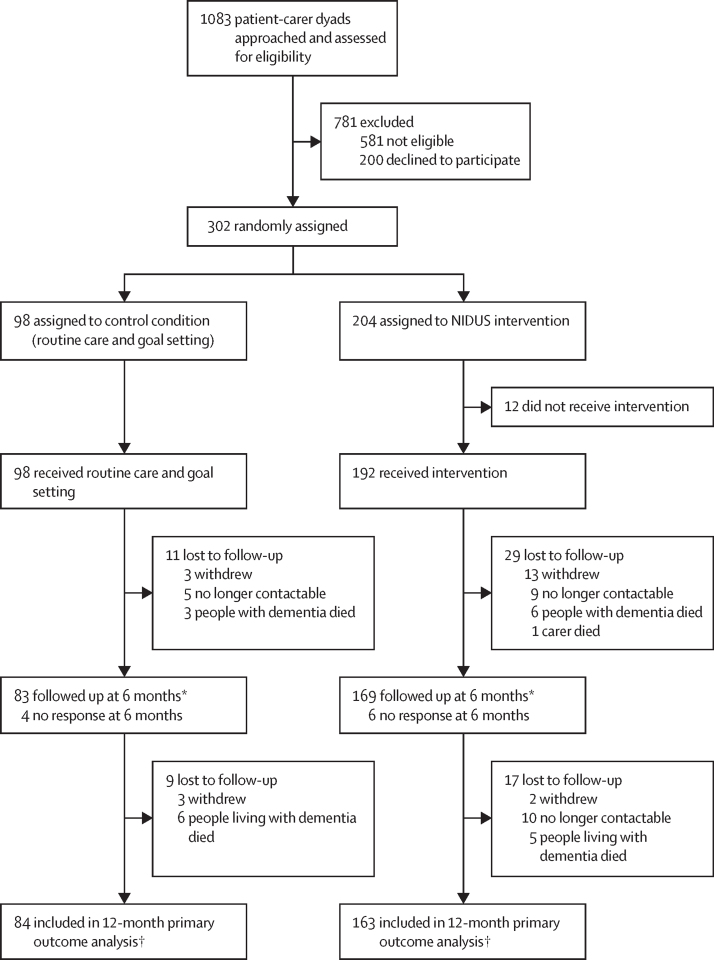
Trial profile GAS=Goal Attainment Scaling. *Numbers are those providing any data at 6 months. For 52 participants (19 in the control group and 33 in the NIDUS-Family intervention group), data provided were for the 6-month GAS outcome only. †GAS was scored and analysed in the first follow-up after death—ie, for people who died between 6 and 12 months, GAS was scored and analysed at 12 months (six in the control group and four in the NIDUS-Family intervention).

Between April 30, 2020, and May 9, 2021, we assessed 1083 potential dyads for eligibility, 781 (72·1%) of whom were excluded (figure 1). Of 302 eligible dyads, we randomly assigned 98 (32·4%) to the control group and 204 (67·5%) to the intervention group (figure 1). 247 (82%) of 302 randomly assigned dyads completed the primary outcome (figure 1). 21 sites recruited a mean of 14 dyads (SD 9; range 3–31). The mean age of participants with dementia was 79·9 years (SD 8·2), 169 (56%) were women, and 133 (44%) were men. 237 (78%) identified as White British, 29 (10%) as White ethnic groups, 17 (6%) as Asian, 11 (4%) as Black, four (1%) as mixed, and four (1%) as other ethnic groups.

Baseline characteristics were similar between allocated groups, although more people with dementia were female and more carers were male in the control group than in the intervention group (Table 1, Table 2). At 12 months, raters correctly guessed 61% of allocations to treatment and 65% of allocations to control (n=235). Only two dyads completed baseline measures in person and one carer completed follow-up measures in person. Although COVID-19 restrictions had lifted in later phases of the trial, many people continued to limit their in-person interactions.

**Table 1:** Baseline characteristics of people with dementia by randomised group

		**Control (n=98)**	**NIDUS-Family intervention (n=204)**	**Total (n=302)**
Age, years		80·3 (8·7)	79·7 (8·0)	79·9 (8·2)
Ethnicity				
	White British	76 (77·6%)	161 (78·9%)	237 (78·5%)
	White other	11 (11·2%)	18 (8·8%)	29 (9·6%)
	Mixed	2 (2·0%)	2 (1·0%)	4 (1·3%)
	Asian	5 (5·1%)	12 (5·9%)	17 (5·6%)
	Black	2 (2·0%)	9 (4·4%)	11 (3·6%)
	Other	2 (2·0%)	2 (1·0%)	4 (1·3%)
First language				
	English	83 (84·7%)	177 (86·8%)	260 (86·1%)
	Other	15 (15·3%)	27 (13·2%)	42 (13·9%)
Sex				
	Male	38 (38·8%)	95 (46·6%)	133 (44·0%)
	Female	60 (61·2%)	109 (53·4%)	169 (56·0%)
Marital status				
	Married or civil partnership	57 (58·2%)	116 (56·9%)	173 (57·3%)
	Divorced	7 (7·1%)	10 (4·9%)	17 (5·6%)
	Widowed	33 (33·7%)	67 (32·8%)	100 (33·1%)
	Single, co-habiting, or other	1 (1·0%)	11 (5·4%)	12 (4·0%)
Education (n=296)				
	Higher degree	9 (9·2%)	24 (12·1%)	33 (11·1%)
	Degree	18 (18·4%)	38 (19·2%)	56 (18·9%)
	A level (or equivalent)	9 (9·2%)	16 (8·1%)	25 (8·4%)
	HNC or HND (or equivalent)	7 (7·1%)	14 (7·1%)	21 (7·1%)
	NVQ (or equivalent)	5 (5·1%)	7 (3·5%)	12 (4·1%)
	GCSE (or equivalent)	17 (17·3%)	30 (15·2%)	47 (15·9%)
	School Leaving Certificate	16 (16·3%)	39 (19·7%)	55 (18·6%)
	No formal qualifications	17 (17·3%)	30 (15·2%)	47 (15·9%)
Living situation				
	Living alone	22 (22·4%)	62 (30·4%)	84 (27·8%)
	Living with partner or spouse	52 (53·1%)	107 (52·5%)	159 (52·6%)
	Living with children	16 (16·3%)	23 (11·3%)	39 (12·9%)
	Other	8 (8·2%)	12 (5·9%)	20 (6·6%)
Had capacity to consent				
	No	64 (65·3%)	114 (55·9%)	178 (58·9%)
	Yes	34 (34·7%)	90 (44·1%)	124 (41·1%)
Co-resident with carer				
	No	31 (31·6%)	78 (38·2%)	109 (36·1%)
	Yes	67 (68·4%)	126 (61·8%)	193 (63·9%)
Accommodation				
	Council rented	5 (5·1%)	15 (7·4%)	20 (6·6%)
	Housing association rented	5 (5·1%)	9 (4·4%)	14 (4·6%)
	Private rented	3 (3·1%)	10 (4·9%)	13 (4·3%)
	Owner occupied	82 (83·7%)	155 (76·0%)	237 (78·5%)
	Other	3 (3·1%)	15 (7·4%)	18 (6·0%)
Dementia diagnosis				
	Alzheimer's Disease	44 (44·9%)	95 (46·6%)	139 (46·0%)
	Vascular dementia	10 (10·2%)	28 (13·7%)	38 (12·6%)
	Lewy body dementia	3 (3·1%)	7 (3·4%)	10 (3·3%)
	Frontotemporal dementia	2 (2·0%)	6 (2·9%)	8 (2·6%)
	Other	26 (26·5%)	58 (28·4%)	84 (27·8%)
	Unable to specify	13 (13·3%)	10 (4·9%)	23 (7·6%)

Data are n (%) or mean (SD). GCSE=General Certificate of Secondary Education. HNC=Higher National Certificate. HND=Higher National Diploma. NVQ=National Vocational Qualification.

**Table 2:** Baseline carer characteristics by randomised group

		**Control (n=98)**	**NIDUS-Family intervention (n=204)**	**Total (n=302)**
Carer age, years		64·0 (11·5)	63·1 (12·9)	63·4 (12·5)
Carer ethnicity				
	White British	75 (76·5%)	157 (77·0%)	232 (76·8%)
	White other	11 (11·2%)	23 (11·3%)	34 (11·3%)
	Mixed	2 (2·0%)	2 (1·0%)	4 (1·3%)
	Asian	5 (5·1%)	11 (5·4%)	16 (5·3%)
	Black	2 (2·0%)	9 (4·4%)	11 (3·6%)
	Other	3 (3·1%)	2 (1·0%)	5 (1·7%)
Carer first language				
	English	88 (89·8%)	189 (92·6%)	277 (91·7%)
	Other	10 (10·2%)	15 (7·4%)	25 (8·3%)
Carer sex				
	Male	38 (38·8%)	52 (25·5%)	90 (29·8%)
	Female	60 (61·2%)	152 (74·5%)	212 (70·2%)
Carer marital status				
	Married or civil partnership	77 (78·6%)	156 (76·5%)	233 (77·2%)
	Divorced	5 (5·1%)	6 (2·9%)	11 (3·6%)
	Single	9 (9·2%)	24 (11·8%)	33 (10·9%)
	Co-habiting	4 (4·1%)	14 (6·9%)	18 (6·0%)
	Widowed	3 (3·1%)	2 (1·0%)	5 (1·7%)
	Other	0	2 (1·0%)	2 (0·7%)
Carer education				
	Higher degree	18 (18·4%)	37 (18·1%)	55 (18·2%)
	Degree	30 (30·6%)	67 (32·8%)	97 (32·1%)
	A level (or equivalent)	11 (11·2%)	31 (15·2%)	42 (13·9%)
	HNC or HND (or equivalent)	10 (10·2%)	8 (3·9%)	18 (6·0%)
	NVQ (or equivalent)	4 (4·1%)	16 (7·8%)	20 (6·6%)
	GSCE (or equivalent)	16 (16·3%)	26 (12·7%)	42 (13·9%)
	School Leaving Certificate	6 (6·1%)	7 (3·4%)	13 (4·3%)
	No formal qualifications	3 (3·1%)	12 (5·9%)	15 (5·0%)
Relationship of carer to person with dementia				
	Spouse or partner	51 (52·0%)	102 (50·0%)	153 (50·7%)
	Child	46 (46·9%)	91 (44·6%)	137 (45·4%)
	Friend	0	1 (0·5%)	1 (0·3%)
	Other	1 (1·0%)	10 (4·9%)	11 (3·6%)
Carer living situation				
	Living alone	4 (4·1%)	9 (4·4%)	13 (4·3%)
	Living with partner or spouse	69 (70·4%)	148 (72·5%)	217 (71·9%)
	Living with housemates	0	2 (1·0%)	2 (0·7%)
	Living with parent	13 (13·3%)	14 (6·9%)	27 (8·9%)
	Living with children	2 (2·0%)	8 (3·9%)	10 (3·3%)
	Other	10 (10·2%)	23 (11·3%)	33 (10·9%)
Carer accommodation				
	Council rented	5 (5·1%)	10 (4·9%)	15 (5·0%)
	Housing association rented	4 (4·1%)	6 (2·9%)	10 (3·3%)
	Private rented	3 (3·1%)	13 (6·4%)	16 (5·3%)
	Owner occupied	85 (86·7%)	169 (82·8%)	254 (84·1%)
	Other	1 (1·0%)	6 (2·9%)	7 (2·3%)

Data are n (%) or mean (SD). GCSE=General Certificate of Secondary Education. HNC=Higher National Certificate. HND=Higher National Diploma. NVQ=National Vocational Qualification.

The ten facilitators supported a mean of 20·4 participating dyads each (SD 13·6; range 4–46). 175 dyads completed at least six sessions, but we defined the full intervention as receipt of at least six sessions including the final session, and one of these dyads did not complete the final session (appendix p 17). 30 (15%) dyads withdrew from the intervention because the person with dementia died (n=6), the dyad did not want to continue (n=10) or became uncontactable (n=7), or because of hospitalisation or other serious adverse events (n=7). Intervention withdrawals occurred across 12 sites and in self-referred dyads. The mean number of drop-outs from these sites was 2·3 dyads (SD 1·3; range 1–5). Dyads received the intervention face-to-face (n=3), by telephone (n=63), or via video call (n=126). 12 (6%) dyads randomly assigned to the intervention did not receive the intervention.

Dyads received a mean of 6·5 sessions (SD 2·3; median 7 sessions [IQR 6–8]) and 1·6 (SD 1·8) telephone follow-up sessions between 6 and 12 months (median 1 session [IQR 0–3]; appendix p 16). Modules relating to managing mood, identifying enjoyable activities, and carer wellbeing and support were most frequently delivered (appendix p 16). Researchers completed fidelity checks on intervention sessions for 35 (17%) dyads receiving the intervention. Overall, researchers strongly agreed that the facilitators kept all 35 dyads engaged. For 34 dyads, researchers agreed or strongly agreed that facilitators kept the dyad focused, and for one dyad researchers neither agreed nor disagreed. For 31 dyads, they agreed or strongly agreed that facilitators kept to time; four checklists indicated the sessions went over time by 10–20 min.

**Figure 2:**
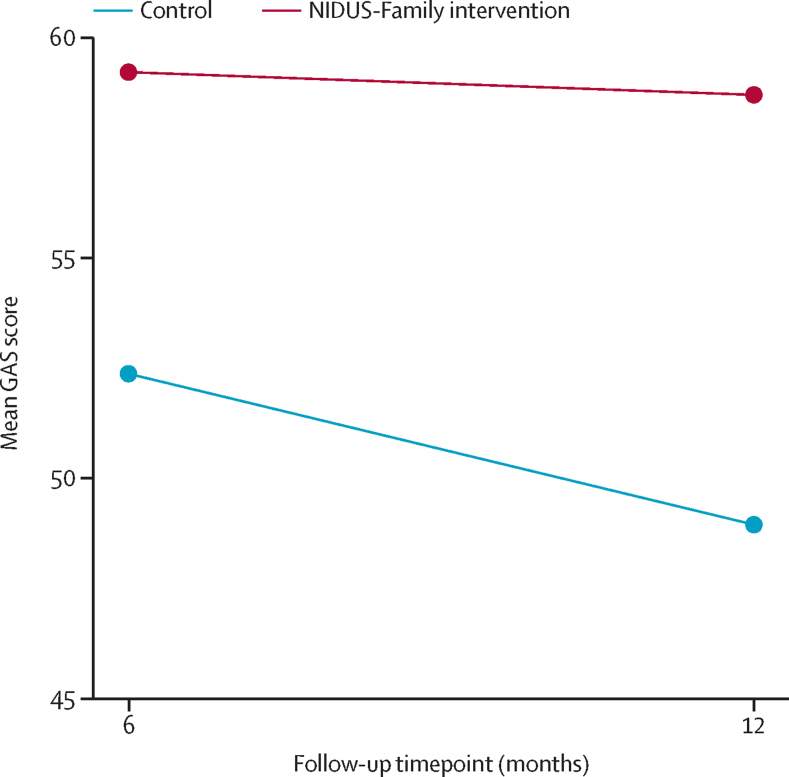
GAS scores at 6 and 12 months by randomised group GAS=Goal Attainment Scaling.

6-month follow-up assessments occurred at a median of 191 days (IQR 181–204; between Oct 6, 2020, and Nov 15, 2022) after baseline, and 12-month follow-ups occurred at a median of 374 days (364–391; between March 26, 2021, and May 29, 2023) after baseline. In analyses accounting for site and facilitator, the mean GAS score at 12 months was 10·23 points (95% CI 5·75–14·71) higher for the intervention group compared with control (58·7 [SD 13·0], n=163, *vs* 49·0 [14·1], n=84; p<0·001; figure 2). This difference equates to a large effect size (Cohen's d=0·75). Dyads set a mean of 3·5 goals (SD 0·6; 3·4 [0·6] in the intervention group and 3·5 [0·7] in the control group). Dyads set 1043 goals in total; 719 (69%) were related primarily to the person living with dementia and 324 (31%) to carer support or wellbeing. In 258 (85%) of 302 dyads, goals were set by the carer and in 44 (15%), goals were set by the dyad together. Goal content is described elsewhere^21^ and summarised in the appendix (p 18).

For the secondary outcome of GAS scores rated by carers at 6 months, and at 6 and 12 months by researchers, scores also favoured the intervention (Table 3, Table 4; appendix p 19). DAD scores favoured the control group (difference in means at 12 months –7·05 [95% CI –13·00 to –1·10]), indicating that individuals in the intervention group were performing a lower proportion of the instrumental activities of daily living that they had an opportunity to attempt in the 2 previous weeks without assistance or reminders compared with those in the control group. No other secondary outcomes differed significantly by group (Table 3, Table 4; appendix p 19). Carer proxy-rated quality of life was 3 points higher in the intervention group than in the control group at 12 months, indicating a small effect size (Cohen's d=0·31) that was not statistically significant (difference in means –2·66 [95% CI –16·19 to 10·87]; p>0·05).

**Table 3:** Secondary outcome results from mixed effects linear regression models

	**N**	Difference in means (95% CI)*
**6 months**		
GAS score (carer rated)	261	6·79 (3·48 to 10·10)†
GAS score (researcher rated)	261	6·63 (3·31 to 9·95)†
DAD score	206	−4·57 (−9·47 to 0·33)
DEMQOL score	46	−2·91 (−10·76 to 4·93)
DEMQOL proxy score	205	0·12 (−3·27 to 3·52)
NPI score	208	0·62 (−3·68 to 4·92)
HADS anxiety score	200	−0·50 (−1·47 to 0·46)
HADS depression score	200	−0·39 (−1·28 to 0·51)
b-DAS executive score	184	−0·13 (−0·61 to 0·36)
b-DAS emotional score	184	−0·11 (−0·66 to 0·44)
**12 months**		
GAS score (researcher rated)	247	10·63 (6·14 to 15·12)†
DAD score	162	−7·05 (−13·00 to −1·10)†
DEMQOL score	26	−2·66 (−16·19 to 10·87)
DEMQOL proxy score	161	3·08 (−1·08 to 7·24)
NPI score	159	0·70 (−5·75 to 7·16)
HADS anxiety score	161	−0·40 (−1·45 to 0·65)
HADS depression score	161	0·03 (−0·85 to 0·91)
b-DAS executive score	148	−0·41 (−0·90 to 0·07)
b-DAS emotional score	148	−0·16 (−0·67 to 0·35)

b-DAS=brief Dimensional Apathy Scale. DAD=Disability Assessment for Dementia. DEMQOL=Dementia Quality of Life. GAS=Goal Attainment Scaling. HADS= Hospital Anxiety and Depression Scale. NPI=Neuropsychiatric Inventory.

* Calculated as intervention – control.

† Significant at 5% level.

**Table 4:** Secondary outcome results from mixed effects ordered logistic regression models

	**n**	Odds ratio (95% CI)*
**6 months**		
b-DAS initiation score	184	1·49 (0·80–2·74)
MCTS score	203	0·75 (0·42–1·36)
**12 months**		
b-DAS initiation score	148	2·44 (0·88–6·72)
MCTS score	157	0·69 (0·34–1·39)

b-DAS=brief Dimensional Apathy Scale. MCTS=Modified Conflict Tactics Scale.

* Intervention compared with the control condition.

We observed 11 adverse events in ten people with dementia (eight in the intervention group and two in the control group; appendix p 25). We observed 50 serious adverse events, all unrelated to the intervention, in 45 people with dementia (31 in the intervention group [15% of participants] and 14 in the control group [14%] of participants; appendix p 25). In the control group, nine people with dementia had died and four had moved permanently to a care home (13 [13%] of 98 known to no longer be living permanently at home), and in the intervention group 11 had died and eight moved permanently to a care home (19 [9%] of 204) by 1-year follow-up (appendix p 25).

Refitted primary models that adjusted for participants’ first language (a predictor of missing values for primary outcome), used imputed missing values, and adjusted for prespecified baseline factors or for imbalances in other baseline factors between groups (sex of carer and of person living with dementia), gave similar findings to those of the primary model (appendix pp 26–28).

We included 270 participant dyads, having at least one GAS measurement (at 6 months or 12 months), in the repeated measures analysis. Estimates of the average difference between groups were 6·93 (95% CI 2·24 to 11·61) at 6 months and 9·99 (5·27 to 14·72) at 12 months. The interaction between randomised group and the relative calendar time of randomisation was not statistically significant (p=0·54). Treatment effects within subgroups were 6·3 (–0·87 to 13·50) among dyads in which people living with dementia had capacity to provide consent, 12·2 (6·48 to 17·84) among dyads in which people living with dementia did not have capacity, 7·3 (1·80 to 12·70) in those with resident carers, and 15·0 (8·23 to 21·74) in those with non-resident carers.

The treatment effect for the primary outcome was 12·1 (95% CI 7·55–16·66) for those receiving the intervention by video or face-to-face and 6·0 (0·70–11·28) for those receiving the intervention by telephone (compared with the control group). These results might be confounded because this analysis was not comparing randomly assigned groups. Secondary outcome models that were adjusted for predictors of missing outcome data and repeated measurements analyses were similar to the main results (appendix pp 22–23).

## Discussion

NIDUS-Family with goal setting effectively improved dyads’ goal attainment, compared with goal setting and routine care, over 1 year. To our knowledge, this is the first intervention delivered by non-clinical facilitators and the first able to be delivered remotely shown to improve goal attainment in people living with dementia. Since we compared the intervention with an active goal-setting control and because setting a goal is often helpful in itself, the reported intervention effect might be underestimated.^20^

The NIDUS-Family approach to post-diagnostic care is novel and we think that focusing care on people's personal priorities is an appropriate way to deliver services. No manualised, and therefore scalable, interventions have been previously shown to improve attainment of personalised goals or functioning in people with dementia.^5^ Secondary outcomes showed no significant differences between intervention and control groups, except for the DAD score, which favoured the control group. A previous trial that reported statistically significant improvements in goal attainment also found improvements were not accompanied by commesurate changes on standardised outcome scales.^9^ We postulate that goal-attainment measures might be more sensitive to clinically important change than generic outcomes and might be a useful approach to assessing and planning post-diagnostic support. This putative explanation of findings is supported by psychometric assessments of GAS,^20^ which have shown GAS has greater sensitivity to change compared with standard measures of functioning, when measured against a patient and a masked physician global measure, as well as the numerically higher average proxy-rated quality of life scores (by 3 points) in the intervention versus control group after 1 year. Although not statistically significant, this effect size is previously reported as a clinically important difference,^29^ but the current trial might not have been sufficiently powered to detect it.

Alternatively, GAS might have been measuring conceptually distinct outcomes to the secondary, generic outcomes, which tend to be rooted in “a deficiency-focused approach, with focus on impairment as opposed to measures capturing strengths, adaptations, resilience, and well-being”.^30^ For example, in the WHO healthy ageing framework, functional ability is described as comprising intrinsic capacity of the individual, of relevant environmental characteristics and of their interaction. The framework seeks to address “a danger that we will continue to just measure intrinsic capacities ignoring surrounding conditions”.^31^ NIDUS-Family aims to increase goal attainment through functional and environmental adaptation and through optimising care. Consistent with this approach, DAD scores showed that intervention participants received assistance or prompting in greater proportions of instrumental activities of daily living than control participants did. The most common type of goal set was to support engagement in activities and, for most dyads, even +2 goals did not anticipate this engagement would be entirely without support. Greater awareness of the person with dementia's functioning among carers in the intervention group, compared with those in the control group, might also have accounted for this finding.

A scoping review,^32^ indicating that GAS is often inadequately reported or used inconsistently, proposes a guideline to support its implementation. As the authors of this previous review advocated, we maximised GAS validity through third-party review of all goals and facilitator training. We asked researchers to rate goal attainment, and we found that these ratings were similar to family-carer ratings. Goal setting is a key component of person-centred care; the ability of non-clinicians to robustly and consistently use GAS is an important finding from this study, for clinical practice and scalability.

Around two-thirds of researchers who completed GAS at 6 and 12 months correctly guessed group allocation, which could indicate some unblinding or could be explained by intervention effectiveness. As is the case in all psychological treatment trials, we could not mask participant dyads to allocation status. The DAD scale is primarily aimed at people in earlier stages of dementia,^33^ and the large proportion of people with moderate and severe dementia included in this study might have limited the score's interpretation. The DAD scale does not capture the level of prompting or support provided, nor the complexity, frequency, or quality of the instrumental activity of daily living performed in each area assessed. We used capacity to consent as a proxy for illness stage but did not measure cognition directly, to minimise burden on participants, and because our intervention was not directly cognition.

In the longer term, NIDUS-Family aims to support people with dementia to live longer in their own homes. A systematic review^6^ identified two interventions that achieved this goal. Both RCTs, done in the US, were delivered by clinically trained staff. One study mapped care needs to a list of interventions, including signposting, psychosocial, and environmental interventions.^34^, ^35^ The other provided needs-tailored counselling to carers.^36^ We will continue to follow our cohort to see whether this longer-term goal of supporting people with dementia to live longer in their homes was achieved. Noting that the difference in goal attainment between groups was greater at 12 months than at 6 months, we hope the strategies planned during the intervention will remain useful beyond the main intervention period.

To ensure NIDUS-Family is accessible for people with more severe dementia, we asked carers to set goals, to which people living with dementia contributed to the extent they were able. We cannot independently verify how goals reflected the wishes of people with dementia who lacked capacity. Social conditions (during and after pandemic-related restrictions) might have affected goal attainment, although our sensitivity analysis adjusting for calendar date found no evidence for this. We did not include people with dementia who did not have a regular carer or whose carer could not access the intervention written in English. We plan to adapt NIDUS-Family for non-English speaking UK populations.

The good adherence rates reported might relate to intervention flexibility, including the participant's involvement in deciding session content, and whether to meet using video call or telephone or, when possible, in person. Two key mechanisms for ensuring that this complex intervention could be delivered by non-qualified staff in the trial were the fully structured facilitator and participant manuals, which drew on evidence-based best practice and were co-produced with experts with professional and lived experience, and regular clinical supervision from experienced and trained clinicians. We have previously published perspectives^37^ of the non-clinical facilitators, who valued regular supervision highly. A process evaluation study that we will publish separately explores mechanisms of action to inform a planned implementation study to commence in 2024. Essential to this implementation will be ascertaining how to scale up training and supervisory support.

We will next explore how to support translation of findings into practice, at an important and hopeful time for dementia treatment and care. New disease-modifying treatments are likely to drive earlier diagnoses and delay disease progression in a proportion of those with Alzheimer's disease who are eligible and in whom new drugs are tolerated. Strengthening the evidence base for non-pharmacological therapies will complement these new drug treatments. As scalable, inclusive, personalised care and support, NIDUS-Family is ready to be implemented alongside new investments in early detection, diagnostic, and drug treatment facilities. NIDUS-Family's approach aligns with aspirations of the NHS Long Term Workforce Plan, announced in June, 2023, to innovate and grow the health-care workforce.^38^ We recommend that post-diagnostic services routinely provide goal-focused, structured, manualised support to all people diagnosed with dementia who have a carer.

## Data sharing

Data collected for the study, including the statistical analysis plan, de-identified participant data, and a data dictionary defining each field in the set, will be made available to others on receipt by Priment CTU (priment@ucl.ac.uk) of a reasonable request, at any date after publication of this paper. All requests will be reviewed by Priment CTU in line with Priment CTU guidance on sharing data and anonymising data. This process is to ensure that the request is reasonable and the data set is suitably anonymised. The study protocol is available open access. Intervention materials are available without cost, subject to a CC BY-NC-ND license held by the corresponding author.

## Declaration of interests

KR reports personal fees (primarily for invited guest lectures, rounds, and academic symposia on frailty) from the Burnaby Division Family Practice, McMaster University, Chinese Medical Association, Wake Forest University Medical School Centre (advisory board member), University of Omaha, the Atria Institute, EPI Pharma (data safety monitoring board advisory board member), and Ardea Outcomes, outside the submitted work. All other authors declare no competing interests.
