# Supplementary material for: A psychosocial goal-setting and manualised support intervention for independence in dementia (NIDUS-Family) versus goal setting and routine care: a single-masked, phase 3, superiority, randomised controlled trial
Source: Lancet Healthy Longev. 2024 Feb;5(2):e141–51. doi: 10.1016/S2666-7568(23)00262-3 (PMC10834374; doi:10.1016/S2666-7568(23)00262-3)
Supplement: Supplementary appendix [file mmc1.pdf]

THE LANCET
Healthy Longevity

Supplementary appendix

This appendix formed part of the original submission and has been peer reviewed. We post it as supplied by the authors.

Supplement to: Cooper C, Vickerstaff V, Barber J, et al. A psychosocial goal-setting and manualised support intervention for independence in dementia (NIDUS-Family) versus goal setting and routine care: a single-masked, phase 3, superiority, randomised controlled trial. *Lancet Healthy Longev* 2024; **5:** e141–51.

NIDUS Family Study

Statistical and Health Economic Analysis Plans

V2.0

Started: 09 December 2021
Edited: 29 March 2023

Version History Log

Version	Date Implemented	Details of change
0.1	09 Dec. 21	
0.2	12 Jan. 22	Including Julie Barber's comments.
0.3	22 Feb. 22	
0.4	01 Mar. 22	
0.5	30 Mar. 22	
0.6	20 Apr. 22	Adding Health Economics to latest version
0.7	7 Jul. 22	Adding supportive analyses for COVID
1.0	12 Jul. 22	
2.0	29 Mar. 23	Adding text relating to collection to outcomes at 24 months

1. Study Summary

For full details see the protocol (version 6 10/11/2021).

**Title**	Clinical and cost-effectiveness of a New psychological intervention to support Independence in Dementia (NIDUS) for family carers and people living with dementia in their own homes: A randomised controlled trial
**Short title**	Supporting independence at home for people with dementia (NIDUS-Family)
**Chief Investigator**	Prof. Claudia Cooper
**Statisticians**	Senior statistician: Dr Julie Barber<br>Trial statistician: Dr Victoria Vickerstaff
**Health economist**	Rachael Hunter
**Design**	An individually randomised single-masked multi-site clinical trial with people living with dementia and their family carers (dyads). Dyads will be randomised 2:1 to receive the NIDUS-family intervention and routine care or routine care alone.
**Primary objective**	To i) evaluate the effect of NIDUS-family and routine care on goal attainment as measured by Goal Attainment Scaling (GAS) rated by family carers (10, 11) compared to routine care alone and ii) determine whether NIDUS-family plus routine care is more cost-effective than routine care alone at 12-month follow up.
**Population**	297 people living with dementia-family carer dyads (594 participants in total)
**Study Type**	Interventional randomised controlled trial
**IRAS Number**	259249

2. Introduction

2.1. Purpose and scope of the statistical and health economic analysis plan

This document describes the main statistical and health economic analyses to be applied to the data from the NIDUS family trial. This Statistical Analysis Plan was written by Victoria Vickerstaff and Julie Barber. The Health Economics Analysis Plan was written by Rachael Hunter.

2.2. Timing of Analysis

The analysis described within this analysis plan will be performed after all the data up to and including the twelve months follow up have been entered, checked and locked and this analysis plan has been finalised.

2.3. Data checking

Before analysis and database lock, basic checks will be performed on the quality of the data, focusing on identifying:

- Missing data
- Data outside expected range
- Other inconsistencies between variables e.g. in the dates the questionnaires were completed

If any inconsistencies are found, the corresponding values will be double checked with the researchers and corrected if necessary in the source data. This checking process and subsequent changes will be documented.

3. Description of the trial

3.1. Duration of the treatment period and frequency of follow up

In the intervention arm, data collection at baseline will be followed by an intervention period lasting for a maximum of 1 year, consisting of six months for delivery of up to eight sessions of the intervention and an additional six -months of telephone follow-ups every six to eight weeks. For both arms, data will be collected at baseline (randomisation) and at six, twelve, 18 and 24 months from randomisation. However, this analysis plan will only discuss the data collected at baseline and six and twelve months. The analysis for the data at 18 and 24 months will be viewed as a secondary analysis.

4. Data collection

4.1. Participant characteristics

The following data will be collected from all participants recruited into the study at the baseline assessment:

Information about the family carer

1. Age, gender, marital status, employment, ethnicity, first language, education and living arrangements

2. Relationship to person with dementia

Information about the person with dementia

1. Diagnosis of dementia
2. Age, gender, marital status, employment, ethnicity, first language, education, living arrangements, if they are in receipt of home care services

4.2. Outcome data

The outcomes will be collected at baseline, six, twelve, 18 and 24 months follow up. However, in this analysis plan we will only discuss the outcomes collected at baseline, six and twelve months.

4.2.1. Primary outcome

The primary outcome is functioning of the person with dementia assessed using family carer rated Goal Attainment Scaling (P-GAS). Family carers will evaluate 'performance' on a minimum of three and maximum of five goals set at baseline, on a 5-point scale ranging from "much worse" to "much better" than expected. As people will have different goals and numbers of goals, a summary formula standardises the degree of goal attainment, analysed as a change score.

4.2.2. Secondary outcome measures

Family carers will be asked to proxy-complete at baseline, six and twelve months follow up all of the following assessments.

- Disability Assessment for Dementia scale, a standard measure of functional independence (basic and instrumental activities of daily living);
- DEMQOL proxy, a widely used measure of quality of life of people with dementia;
- Neuropsychiatric inventory;
- Carerqol, measures of family carer quality of life;
- Brief Dimensional Apathy Scale;
- Hospital Anxiety and Depression Scale;
- Modified Conflict Tactics Scale to measure potentially abusive behaviours;
- Client Services Receipt Inventory (CSRI) including home care, hospitalisations, respite and all-cause time to transition from home.

People living with dementia (Plwd) will complete the following assessments at baseline, six and twelve months follow up, if they are able to.

- DEMQoL to rate their quality of life

Researchers will be asked to complete at six and twelve months follow up:

- Functioning of the person with dementia using Goal Attainment Scaling (C-GAS).

Details of the outcome measures are provided in the table below.

Outcome	Details and scoring
Goal Attainment Scaling (GAS) (Rockwood et al., 2002; Rockwood et al., 2003)	GAS is a method of scoring the extent to which patient's individual goals are achieved as measured by family carers (P-GAS) and researchers (C-GAS). Family carers and researchers will evaluate the 'performance' on a minimum of three and maximum of five goals set at baseline, on a 5-point scale ranging from "much worse" to "much better" than expected. As people will have different goals and numbers of goals, a summary formula will be used that standardises the degree of goal attainment, analysed as a change score. The summary formula is:<br><br>$$T = 50 + \frac{10\ \Sigma x_i}{\sqrt{(1-\rho)n + \rho n^2}}$$<br><br>where<br>x_i is the degree to which the goal is achieved [-2, 2]<br>ρ is the expected overall inter-correlation between goal areas (typically 0.3)<br>n is the number of goals $i = 1, \ldots, n$<br><br>The score can be interpreted as follows:<br>T = 50 = met baseline expectations (no change)<br>T < 50 = did not meet baseline expectations (worsened)<br>T > 50 = exceeded baseline expectations (improved)
Disability Assessment for Dementia (DAD) scale (Feldman et al., 2001)	The DAD measures functional abilities in activities of daily living (ALD) in individuals with dementia. The measure contains 40 items. Each item can be scored: Yes = 1 point, No = 0 points or non applicable = N/A.<br><br>A total score is obtained by adding the rating for each question and converting this total score out of 100. The items rated as N/A are not considered for the total score.<br><br>For example:<br>A score of 33 on 40 (maximum score) converted out of 100 = 83%<br>A score of 33 on 38 (max. score with 2 N/A) converted out of 100= 87%<br><br>Higher scores represent less disability in ADL while lower scores indicate more dysfunction.
DEMQOL (Banerjee et. al, 2004)	DEMQOL is a 29 item questionnaire answered by the person with dementia. It is designed to enable the assessment of health-related quality of life for people with dementia. Each item can be scored as: A lot = 1; Quite a bit = 2; A little = 3; Not at all = 4<br>Positive items need to be reversed so that for all items a higher score means better quality of life. Items: 1, 3, 5, 6, 10 and 29 need reversing.<br>The overall DEMQOL score is computed by adding up items 1-28 and provides a score between 4 and 112, with higher scores indicating better quality of life.<br><br>As recommended for DEMQoL calculation, where less than 50% of data items are missing, these items will be imputed using the within participant mean.

<table>
<tr><td>Outcome</td><td>Details and scoring</td></tr>
<tr><td></td><td></td></tr>
<tr><td>DEMQOL proxy (Banerjee et. al, 2004)</td><td>DEMQOL-Proxy is a 32 item questionnaire answered by a caregiver. Data should be entered as: A lot = 1; Quite a bit = 2; A little = 3; Not at all = 4
Positive items need to reversed so that for all items a higher score means better quality of life. Items: 1, 4, 6, 8, 11 and 32 need reversing.
The overall DEMQOL score is computed by adding up items 1-31.

As recommended, where less than 50% of data items are missing, these items will be imputed using the within participant mean.</td></tr>
<tr><td>Neuropsychiatric inventory (Cummings, 1997)</td><td>The NPI assesses dementia-related behavioural symptoms. The measure has 12 domains. Higher scores indicate worse symptoms.
The questionnaire provides frequency, severity and distress ratings for each domain. A frequency, severity, total (frequency x severity) and distress score can be calculated by adding the scores for each domain together. This study will use the total NPI score. The total NPI score ranges from 0 to 144.</td></tr>
<tr><td>CarerQol (Hoefman, van Excel and Brower, 2013)</td><td>CareQol measures family carer quality of life and is designed to measure and value the impact of providing informal care on carers. The measure has seven items, each with three responses (no, some, a lot). We will use the utility tariffs shown below to calculate a weighted sum score of the CarerQol-7D.
Using the weighted sum score, the worst caregiving situation receives a score of 0, while the best has a score of 100.

The analysis of this outcome measure will be described in the health economic plan.
<table>
<tr><td>Dimension</td><td colspan="3">Tariff for score</td></tr>
<tr><td></td><td>No</td><td>Some</td><td>A lot</td></tr>
<tr><td>Fulfilment</td><td>0.0</td><td>10.1</td><td>13.0</td></tr>
<tr><td>Relational problem</td><td>12.0</td><td>8.8</td><td>0.0</td></tr>
<tr><td>Mental health problems</td><td>23.9</td><td>16.5</td><td>0.0</td></tr>
<tr><td>Problems combining daily activities</td><td>7.4</td><td>3.1</td><td>0.0</td></tr>
<tr><td>Financial problems</td><td>17.2</td><td>11.5</td><td>0.0</td></tr>
<tr><td>Support</td><td>0.0</td><td>6.9</td><td>8.1</td></tr>
<tr><td>Physical health problems</td><td>18.4</td><td>15.0</td><td>0.0</td></tr>
</table></td></tr>
<tr><td>Brief Dimensional Apathy Scale (Radakovic et. al, 2019)</td><td>The b-DAS is composed of 9 items, equally distributed over Executive, Emotional and Initiation apathy subscales.

Each subscale has a minimum score of 0 (least apathy) maximum score of 9 (most apathy), with clinical cut-offs for Executive > 4, Emotional > 5 and Initiation > 6 apathy subscales. This study will use the three subscale score.</td></tr>
<tr><td>Hospital Anxiety and Depression Scale (HADS) (Zigmond and Snaith, 1983)</td><td>The HADS is a 14-item measure used to assess anxiety and depression symptoms in patients. Of the 14 items, 7 relate to anxiety and 7 relate to depression. Each question is scored from 0 to 3. The HADS produces three total scores: HADS-T an overall total score, HADS-A for anxiety and HADS-D for depression. The totals are calculated by summing all the items, the anxiety items only and the depression items only respectively.</td></tr>
</table>

Outcome	Details and scoring
	We will also dichotomised anxiety and depression scores into 'case' and 'non-case,' with a cut-off point of greater or equal to 9
Client Services Receipt Inventory (CSRI) (Beecham and Knapp, 1992)	The CSRI covers services used. The analysis of this outcome measure will be described in the health economic plan.
Modified Conflict Tactics Scale (MCTS) (Cooper et. al, 2010)	The MCTS measures potentially abusive behaviours by carers towards care recipients. The measure contains 10 items: five psychological abuse items and five physical abusive items, which are scored on a Likert scale from 0 (never) to 4 (all the time).<br><br>The psychological abuse items ask whether they had: (1) screamed or shouted at the CR, (2) used a harsh tone of voice, insulted, sworn at, or called them names, (3) threatened to send them to a care home, (4) to stop taking care of or abandon them, and (5) threatened to use physical force on them.<br><br>The physical abuse items are: (1) whether the carers had been afraid they might hit or hurt them, (2) whether they had withheld food, (3) whether they had hit or slapped them, (4) whether they had shaken, or (5) handled them roughly in other ways.<br><br>A score of ≥2 (sometimes) on any item will be used as a threshold to indicate significant abuse. We will report the proportion of carers meeting abuse "caseness" criteria for any abuse, psychological and physical abuse.<br><br>We will also calculate a total MCTS score by summing all the items.

4.3. Fidelity

To assess fidelity of delivery of NIDUS-Family, checklists will be applied independently to a random sample of 20% of the transcribed audio recordings of appointments by two researchers.

4.4. Acceptability

Family carers and Plwd will be asked, post intervention (12 months), to rate the acceptability of the intervention on a 5-point Likert scale.

5. Data analysis plan

The statistical analyses will be conducted according to ICH E9 and following the standard operating procedures of the PRIMENT clinical trials unit. All analyses will be carried out using STATA version 17 (or above). The primary analysis will be performed independently by two statisticians (Julie Barber and Victoria Vickerstaff) to ensure its accuracy. The other analyses will be carried out by Victoria Vickerstaff and checked by Julie Barber. Analyses will be carried out based on the intention to treat principle, comparing the groups as randomised regardless of compliance with the intervention. The

primary analysis will be performed on observed outcome values (without imputation, except missing item imputation discussed below to enable us to calculate total scores).

5.1. Recruitment and representativeness of recruited patients

A consort diagram will be constructed to describe the flow of subjects through the trial (http://www.consort-statement.org/). The diagram will detail the number of subjects: approached and assessed for eligibility; eligible; agreeing to enter the study (with reasons for refusal); receiving the intervention (with reason for not receiving this); followed up and withdrawn (with reasons).

5.2. Baseline characteristics

Baseline characteristics of the people living with dementia and their families will summarised by treatment group to gauge the balance in characteristics between the randomised groups. The results will be presented as means with standard deviations for continuous, symmetric variables; medians and inter-quartile ranges for continuous, skewed variables; and frequencies and percentages for categorical variables. No significance testing will be used.

5.3. Adherence to treatment, attrition and missing data

Adherence – numbers of sessions received by participants will be tabulated and reasons for early stopping or missed sessions summarised.

Attrition - Some loss to follow-up is expected over twelve months. Reasons for missing outcome data will be described and frequency (%) of subjects with missing data, by reason will be provided for each randomised group (and for each outcome).

5.4. Adverse event reporting

Adverse events (AE) and serious adverse events (SAE) will be summarised. These events are defined in section 9 of the protocol.

5.5. Analysis of primary outcome

Family carer-rated Goal Attainment Scaling (P-GAS) global mean scores at 12 month follow up will be summarised for the NIDUS-family and treatment as usual groups using means with standard deviations.

The primary analysis will compare the P-GAS global scores between the groups using a three level mixed effects models which allows for intervention arm therapist clustering and also includes a random effect for study site. The treatment effect estimate from this model will be the adjusted difference in mean P-GAS score which will be reported with a 95% confidence interval and P-value.

We will fit the following model (Candlish et. al, 2018):

$$Y_i = \beta_0 + \beta_T T_i + u_{site(i)} + u_{th(i)} T_i + \epsilon_i T_i + \xi_i (1 - T_i)$$

where

Y_i is the outcome (P-GAS global mean score at 12 months)

T_i is the dummy variable for the intervention arm (=0, 1)

β_T is the parameter of interest, adjusted difference between trial arms

i is the participant subscript

$u_{site(i)}$ is a random effect at the study site level

$u_{th(i)}$ is a random effect at the therapist level

ϵ_i is the residual at the participant level

We will use adjusted degrees of freedom (kenward-roger) and restricted maximum likelihood procedure (REML) for estimation, as recommended (Candish et al 2018).

Example STATA code: mixed outcome trgrp || site: || cluster:ttgrp, nocons reml residuals(independent, by(trgrp)) dfmethod(kroger)

If the model does not converge, we will initially try a model with homoscedastic residuals (*Example STATA code:* mixed outcome trgrp || site: || cluster:trgrp, nocons reml dfmethod(kroger)). If there are still convergence issues, we will include study site in the model as a fixed rather than random effect. If convergence issues remain, we will remove the therapist clustering effect as previous studies have found little to no clustering effect for the therapists.

Analysis will compare groups defined by intention to treat and include all those with available data.

5.6. Analysis of secondary outcomes

Scores measured at baseline, six and twelve months will be summarised. We will use mean and standard deviation for continuous variables; adding medians and inter-quartile ranges for continuous, skewed variables; and frequencies and percentages for categorical variables.

Inferential analyses of the secondary outcomes at 6 and 12 months will take a similar approach to the analysis described for the primary outcome, with treatment effect estimates obtained from appropriate mixed effects regression models allowing for therapist clustering (with heteroscedastic residuals) and including a random effect for study site. We will use linear models for continuous secondary outcomes at 6 and 12 months: Disability Assessment for Dementia scale, DEMQOL, DEMQOL Proxy, Neuropsychiatric inventory, Hospital Anxiety and Depression Scale – Anxiety subscale and Depression subscale, MCTs and GAS at 6 months. However, different approaches for analyses of these outcomes will be considered if substantial departures from normality occur (see 'Model Checking' section below). Models for continuous outcomes will additionally include adjustment for the associated baseline measurement, where available.

For the brief Dimensional Apathy Scale we will focus on the three subscales (executive, emotion and initiation). Depending on the distribution of the subscales we will either use linear regression models or ordinal regression models (using the stata command '*meologit*').

The results for the secondary outcomes will be presented as estimates with 95% CIs. P-values will not be reported. Analyses will compare groups defined by intention to treat and include all those with available data.

5.7. Missing items in scales and subscales of secondary outcomes

Missing value guidance is noted for DEMQOL and DEMQOL proxy in the outcome measures table above.

For the other outcome measures, there is no guidance regarding missing items. We will explore the amount of missing data and if there are not too many responses with partially complete responses (<10%) we will consider using individual mean imputation for partially completed responses to enable us to calculate the total scores. If appropriate, we will use individual mean imputation if 10% or fewer items are missing for an individual in a questionnaire. For example, in a scale with 20 items, imputation will be applied to individuals with up to 2 items missing. The average value for the complete items will be calculated for that individual and used to replace the missing values. The scale score will be calculated based on the complete values and these replacements.

5.8. Sensitivity and other planned analyses

Sensitivity analyses for missing outcome data

Under the assumption data are MAR, 2 approaches will be taken for the primary outcome:

1) We will refit models to obtain estimates adjusted for variables associated with missingness. To identify predictors of missing data, characteristics of participants with and without missing outcome data will be compared using logistic regression models (with missing yes/no as the outcome). The main analysis model will be refitted to adjust for any characteristics found to be associated with missingness and the outcome of interest.
2) We will use multiple imputation methods. The imputation model will include the outcome of interest, socio-demographic baseline data and any other variables possibly related to missingness and the outcome. The imputations will be performed by study arm. We will use the number of imputations that is around the proportion of missingness (e.g. 20 imputed sets for 20% missing data) and combine the result using Rubin's rules.

We expect the majority of missing GAS scores to occur for participants who either are admitted to a care home, have died and the death cannot be classified as unexpected or the carer does not wish to score it. Sensitivity analysis where missing values are considered MNAR (those who have been admitted to a care home or have known to have died) will be conducted based on a worst-case scenario. For these participants the follow-up GAS performance scores for each goal will initially be imputed with a value of -2 (representing the participants getting "much worse" than expected). We will re-fit the model using these imputed scores. We will then repeat the analysis assuming a performance value of -1 for these participants.

Similar sensitivity analyses will be considered for secondary outcomes with concerning amounts of missing data.

Supportive analyses

The following supportive analyses will be carried out for the primary and secondary outcomes using the same modelling approaches as described previously:

- Adjusted analyses allowing for the following baseline factors:

    - Recruitment time, (measured in days from the date the first participant was randomised in the study)
    - Level of functioning (measured using the total DAD score at baseline)
    - Carer stress (measured using the total HADS score at baseline)
- Adjusted analyses allowing for the baseline factor recruitment time and an interaction between the randomisation group and recruitment time
- Estimation of the treatment effect adjusting for any concerning imbalances in baseline characteristics.

We will use mean imputation to impute any missing data for the baseline variables required for the above supportive analyses, that is we will impute missing total DAD scores at baseline, total HADS scores at baseline and any baseline characteristics that are imbalanced.

Analysis of repeated measurements of outcome

We will use a mixed effects model based on all participant outcome data over 12 months to investigate how the primary and secondary outcomes change over time. Such models allow analysis of repeated outcome measurements data (recorded at 6 and 12 months) while taking into account the correlation between measurements from the same participant.

We plan to run two models: the first model will be similar to the modelling approach described previously but will additionally account for time; the second model will also include an interaction between the randomisation group and time. By using interaction terms, we will investigate the differences between groups over time. Based on the second model we will also obtain an estimate of the difference between groups at both 6 and 12 months (under MAR assumptions).

Subgroup analyses

In order to explore heterogeneity (or otherwise) of the intervention effect, we will examine the treatment effect across the following:

- Consent provided by Plwd (yes/no)
- Dyads are co-resident (yes/no)
- Mode of delivery (phone call / zoom or face to face)

The estimates of the intervention effect in each subgroup will be shown in a forest plot. The results from these analyses will be treated as exploratory.

5.9. Model checking

The models assume that the residuals are normally distributed and homoscedastic. This will be checked using residual plots. If substantial departures from normality occur, a transformation of the outcome variable will be considered. We will also investigate whether there are any outliers or observations with high leverage.

5.10. Assessment of fidelity to the intervention

The research team will investigate fidelity of delivery of NIDUS-Family. A mean fidelity score will be produced by dividing the number of items on the checklist identified as being delivered in the appointment, by the number of items on the checklist that should have been delivered per appointment, per researcher and across all appointments. We will adopt thresholds used in other intervention fidelity work: where 81–100% constitutes high fidelity, 51-80 is moderate fidelity and 50% or lower constitutes low fidelity.

5.11. General statistical considerations

All statistical tests and confidence intervals will be 2-sided. Confidence intervals will be at the 95% level.

6. Health economic plan

6.1 Aim

The primary aim of the analysis is to calculate the incremental cost per Quality Adjusted Life Year (QALY) gained with the NIDUS-family intervention compared to Treatment as Usual (TAU) alone over 12 months from a health and social care cost perspective.

The secondary aim of the analysis will be to calculate the mean incremental cost per QALY gained over 12 months from a wider societal perspective.

6.2 Outcomes

6.2.1 DEMQOL

The DEMQOL (Banerjee et. al, 2004) is a measure designed to assess the health-related quality of life of people with dementia. We will collect both the DEMQOL and the DEMQOL proxy. Scoring for both is described in section 4.2.2.

6.2.2 Client Service Receipt Inventory (CSRI)

The CSRI is completed by carers at baseline, 6 and 12 months. The CSRI asks questions about services used by the family member including community-based services, Emergency services, inpatient and outpatient hospital visits and any community groups or day centres attended as well as medication use. The CSRI also covers any adaptations that have been made to the participants home and any help they have received with activities such as cleaning, whether state funded, paid out of pocket or unpaid help. Finally, some questions are included to capture the carer's employment and if they have had to take any time off work to care for their relative with dementia or have used any care support services.

We will report descriptive statistics for the percentage of participants using each item in the CSRI as a proportion of their treatment and the mean number of contacts by group for those with none zero contacts at each time point.

6.3 Cost data

6.3.1 Cost of NIDUS-Family

The cost of the intervention will include the cost of training, supervision and staff time to deliver NIDUS-family intervention costed at the relevant staff grade. The total cost of training and supervision

will be divided by the number of participants in the treatment arm to produce a unit cost per participant for training and supervision. We will report the mean cost and standard deviation per participant in the treatment group.

6.3.2 Cost of Health and Social Care Resource Use

The cost of health and social care resource use for the NIDUS-family intervention group vs TAU only will be calculated using the information from the completed CSRI. These will be calculated for each participant using published unit costs from the most recent version of the Unit Costs of Health and Social Care by the Personal Social Services Research Unit (PSSRU) and NHS reference costs. Medication costs will be calculated using the British National Formulary (BNF) costs.

Mean (SD) cost per participant will be reported by group as total cost per participant and by service use type. The difference in health and social care costs between the NIDUS-family group and TAU only group will be reported. Mean incremental cost will be calculated using a three level mixed effects model in line with the statistical analysis plan including a random effect for study site and clustering for therapist effects. We will report the mean difference and bootstrapped 95% CI.

6.3.3 Wider Societal Costs

The wider societal perspective analysis will include out of pocket costs, unpaid help from family/friends, carer time taken off work to care for their relative and any voluntary care services. The average time spent per month on unpaid help will be calculated from responses to the CSRI on unpaid carer time for different activities. Unpaid help will be costed by using the replacement method, multiplying unpaid carer time by how much the unpaid service would have costed if it had been paid for (hourly cost of a home care worker). Unpaid carer time off work will be costed using the human capital approach.

6.4 Quality-Adjusted Life Years (QALYs)

QALYs will be calculated based on participant and family carer responses to the DEMQOL/DEMQOL proxy using the DEMQOL-U/DEMQOL-U-proxy classification system (Mulhern et al., 2013). QALYs will be calculated as the area under the curve adjusting for baseline differences. Mean utility value and mean unadjusted QALYs from baseline to 12 months will be reported for both groups.

The mean incremental difference In QALYs will be calculated using a three level mixed effects model in line with the statistical analysis plan including a random effect for study site and clustering for therapist effects. This will be reported with bootstrapped 95% CI.

6.5 Discounting

No discounting will be used as the time horizon is 12 months.

6.6 Primary Analysis

The primary analysis will use a non-parametric 2-stage bootstrap (TSB) to account for the relationship between costs and outcomes (Gomes et al., 2012).

Incremental cost-effectiveness ratio (ICER)

We will report the mean incremental cost per QALY gained between the NIDUS-family arm and TAU only arm at 12 months. Costs will be bootstrap adjusted as specified above and will include the cost f

health and social care resource use in both arms, the cost of the NIDUS-family intervention will be included for the intervention arm only.

Cost-effectiveness acceptability curve (CEAC) and cost-effectiveness plane (CEP).
The bootstrapped means and 95% CIs for costs and QALYs will be used to calculate the probability that the NIDUS-family intervention is cost-effective compared with TAU only for a range of cost-effectiveness thresholds for one QALY gained. A cost-effectiveness plane will show the bootstrapped results.

6.7 Missing Data

In line with the statistical analysis plan, we will investigate predictors of missingness. If any are statistically significant, we will include them in a sensitivity analysis to restore the assumption of missing at random.

If there is a substantial amount of missing data, we will consider multiple imputation for health economic outcomes as recommended by Faria et al (2014).

6.8 Sensitivity Analysis

We will conduct sensitivity analysis to test the impact of changing assumptions around the cost of the NIDUS-family intervention such as:

- The frequency of supervision and number of researchers per supervision group
- The number of sessions delivered
- Whether sessions are delivered online, in the participant's home or at the researcher's place of work.

6.9 Secondary Analysis

6.9.1 Cost-effectiveness from a wider societal perspective

We will report the ICER, CEP and CEAC for the NIDUS-family intervention vs TAU only at 12 months from a wider societal perspective using the methods described in the primary analysis but including wider societal costs on top of health and social care resource use for both groups.

7. References

Banerjee, S., et al., DEMQOL - Evaluation of a new system for measuring quality of life in people with dementia: Validity, reliability and use in regular practice. Neurobiology of Aging, 2004. 25: p. S321- S321.

Beecham, J. and M. Knapp. Costing psychiatric intervention, in Measuring mental health needs, 1992. C. Thornicrost, C. Brewin, and J. Wing, Editors. London: Gaskell.

Candlish, J., Teare, M.D., Dimairo, M., Flight, L., Mandefield, L. and Walters, S.J., 2018. Appropriate statistical methods for analysing partially nested randomised controlled trials with continuous outcomes: a simulation study. BMC medical research methodology, 18(1), pp.1-17.

Cooper, C., Selwood, A., Blanchard, M., Walker, Z., Blizard, R., & Livingston, G. The determinants of family carer's abusive behaviour to people with dementia: results of the CARD study. Journal of Affective disorders, 2010. 121 (1-2), 136-142.

Cummings, J.L., The Neuropsychiatric Inventory: assessing psychopathology in dementia patients. Neurology, 1997. 48(5): p. 10S-116.

Faria R, Gomes M, Epstein D, White IR. A guide to handling missing data in cost-effectiveness analysis conducted within randomised controlled trials. Pharmacoeconomics. 2014;32(12):1157-70.

Feldman, H., et al., The disability assessment for dementia scale: a 12-month study of functional ability in mild to moderate severity Alzheimer disease. Alzheimer Dis Assoc Disord, 2001. 15(2): p. 89-95.

Gomes M, Ng ES, Grieve R, Carpenter J, Thompson SG. Developing Appropriate Methods for Cost-Effectiveness Analysis of Cluster Randomized Trials. 2012;350–61.

Hoefman, R.J., J. van Exel, and W.B. Brouwer, Measuring the impact of caregiving on informal carers: a construct validation study of the CarerQol instrument. Health Qual Life Outcomes, 2013. 11: p.
173.

Mulhern, B., Rowen, D., Brazier, J., Smith, S., Romeo, R., Tait, R., Watchurst, C., Chua, K.C., Loftus, V., Young, T., Lamping, D., Knapp, M., Howard, R., & Banerjee, S. Development of DEMQOL-U and DEMQOL-PROXY-U: generation of preference-based indices from DEMQOL and DEMQOL-PROXY for use in economic evaluation. Health Technol Assess. 2013. 17(5):v-xv, 1-140.

Radakovic, R., McGrory, S., Chandran, S., Swingler R., Pal, S., Stephenson, L., Colville, S., Newton, J., Starr, J. J., & Abrahams, S. The brief Dimensional Apathy Scale: A short clinical assessment of apathy. *The Clinical Neuropsychologist* doi: 10.1080/13854046.2019.1621382

Rockwood, K., et al., Responsiveness of goal attainment scaling in a randomized controlled trial of comprehensive geriatric assessment. J Clin Epidemiol, 2003. 56(8): p. 736-43.

Rockwood, K., J.E. Graham, and S. Fay, Goal setting and attainment in Alzheimer's disease patients treated with donepezil. J Neurol Neurosurg Psychiatry, 2002. 73(5): p. 500-7.

Zigmond, A.S. and R.P. Snaith, The Hospital Anxiety and Depression Scale. Acta Psychiatrica Scandinavica, 1983. 67(6): p. 361-370.

Figure 1S: The frequency with which different modules of NIDUS-family were delivered

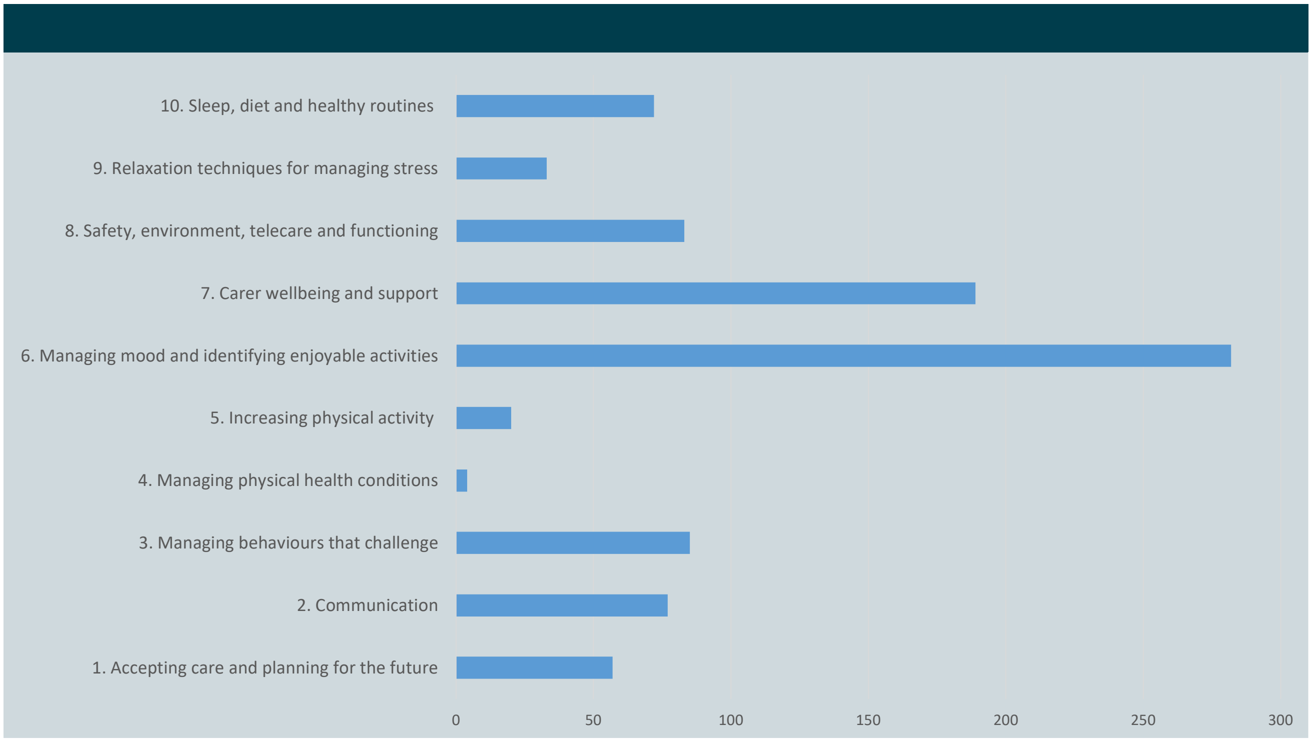

Table 1s Adherence to intervention (n=204)

	n	(%)
**Number of sessions**		
**0**	**12**	**(5.8)**
**1**	**5**	**(2.5)**
**2**	**3**	**(1.5)**
**3**	**5**	**(2.5)**
**4**	**3**	**(1.5)**
**5**	**1**	**(0.5)**
**6**	**37**	**(18.1)**
**7**	**37**	**(18.1)**
**8**	**101**	**(49.5)**

Table 2S: Summary of content of goals set as part of primary outcome

Goal Domain (n, % all 1043 goals)	Goal Area	Number of goals	Example of unmet need/problem
**Mood 245 (23%)**	Improving interest/ initiative	168	PLWD is not engaging in activities that provide mental stimulation
	Reducing anxiety/ worry	45	PLWD is tense and stressed
	Reducing low mood	32	PLWD is easily tearful/upset/crying
**Behaviour 242 (23%)**	Increasing positive interaction with others	120	PLWD has limited interaction with others including family and friends
	Reducing or coping with aggression, irritability, frustration, restlessness, shadowing, repetitive behaviour, or unsafe actions	73	PLWD is quick to anger during conversations with carer ('flies off the handle easily'); or PLWD does not like being left alone more than a few minutes
	Improving sleep disturbances	49	PLWD is waking up distressed in the night
**Self-care 155 (15%)**	Reducing physical complaints	84	PLWD has very limited walking ability
	Improving personal care or dressing	33	PLWD becomes distressed during personal care
	Improving oral intake	38	PLWD is eating less
**Cognition 47 (5%)**	Ability to participate in hobbies/ games	21	PLWD shows no interest in anything and is easily distracted
	Coping with/ improving impact of cognitive decline	26	PLWD cannot remember if they have taken their medication or not
**IADL/ADL 30 (15%)**	Meal Preparation/ Cooking	13	PLWD no longer helps
	Household Chores	12	PLWD no longer helping clean
	Operating Gadgets /Appliances	5	PLWD not able to use new kitchen appliances since moving house
**Carer Break 138 (13%)**	Carer has more time for other activities	123	Carer has no time for sociable activities
	Accessing more care support/overnight respite	15	The carer needs more help with caring (family or professional)
**Carer Mood 99 (10%)**	Reducing carer worry about the future	47	Carer is worried about the future care of PLWD
	Reducing carer worry anxiety	39	Feelings of panic when caring
	Reducing carer low mood	13	Carer is easily tearful and upset
**Carer Behaviour 79 (8%)**	Improving carer reaction to PLWD	57	Irritability/frustration at PLWD behaviour
	Carer accessing more resources	22	Carer does not have enough support
**Carer Sleep 8 (1%)**	Reducing carer sleep problems due to worry/PWLD behaviour	8	PLWD wakes up carer during nights

PLWD: People Living With Dementia

Table 3S Secondary outcomes across the 12 months, by arm									
Visit:	Baseline			6 Months			12 Months		
	N	Mean or n	(SD) or (%)	N	Mean or n	(SD) or (%)	N	Mean or n	(SD) or (%)
GAS participant									
Routine care	.	.	.	86	52.4	(12.6)	84	49.0	(14.1)
NIDUS intervention	.	.	.	175	59.2	(11.3)	163	58.7	(13.0)
GAS researcher									
Routine care	.	.	.	86	52.4	(12.7)	84	48.7	(14.2)
NIDUS intervention	.	.	.	175	59.0	(11.3)	163	58.9	(12.9)
b-DAS total									
Routine care	95	17.2	(2.6)	62	17.4	(2.8)	58	17.8	(2.4)
NIDUS intervention	191	16.6	(3.0)	133	17.3	(2.9)	96	17.4	(2.4)
b-DAS executive									
Routine care	95	3.8	(1.3)	62	3.9	(1.6)	58	3.9	(1.5)
NIDUS intervention	191	3.8	(1.4)	133	3.8	(1.4)	96	3.6	(1.4)
b-DAS emotional									
Routine care	95	5.7	(1.6)	62	6.1	(1.5)	58	6.2	(1.3)
NIDUS intervention	191	5.6	(1.6)	133	5.9	(1.6)	96	6.0	(1.5)
b-DAS initiation									
Routine care	95	7.7	(1.4)	62	7.4	(1.8)	58	7.7	(1.4)
NIDUS intervention	191	7.2	(2.1)	133	7.5	(1.8)	96	7.8	(1.7)
b-DAS executive case (>4), n(%)									
Routine care	95	70	(73.7)	62	41	(66.1)	58	41	(70.7)
NIDUS intervention	191	131	(68.6)	133	98	(73.7)	96	69	(71.9)

b-DAS emotional case (>5), n(%)=1									
Routine care	95	38	(40.0)	62	21	(33.9)	58	16	(27.6)
NIDUS intervention	191	85	(44.5)	133	44	(33.1)	96	35	(36.5)
b-DAS initiation case (>6), n(%)									
Routine care	95	78	(82.1)	62	47	(75.8)	58	46	(79.3)
NIDUS intervention	191	140	(73.3)	133	104	(78.2)	96	75	(78.1)
DAD									
Routine care	97	50.3	(28.3)	66	50.1	(31.7)	59	45.4	(27.8)
NIDUS intervention	204	55.4	(27.0)	140	47.6	(29.5)	103	40.7	(28.7)
DEMQOL									
Routine care	36	88.2	(10.8)	14	92.7	(10.2)	8	91.1	(11.5)
NIDUS intervention	91	90.2	(13.4)	35	88.9	(14.5)	19	91.2	(10.7)
DEMQOL proxy									
Routine care	97	88.7	(14.6)	64	94.0	(13.6)	59	90.4	(13.3)
NIDUS intervention	204	89.9	(14.6)	141	94.1	(13.4)	103	94.8	(15.5)
NPI total									
Routine care	98	27.9	(21.4)	66	22.7	(18.7)	59	23.3	(17.5)
NIDUS intervention	204	24.2	(17.0)	142	22.1	(18.9)	100	23.6	(20.1)
HADS Anxiety									
Routine care	98	7.1	(4.4)	65	6.8	(4.5)	59	6.7	(4.3)
NIDUS intervention	203	6.9	(4.3)	136	6.4	(4.2)	102	6.6	(4.1)
HADS Depression									
Routine care	98	5.4	(4.2)	65	5.4	(4.1)	59	4.8	(3.6)
NIDUS intervention	203	5.0	(3.7)	136	4.8	(3.6)	102	4.7	(3.4)

HADS Anxiety Case (>=9), n(%)									
Routine care	98	38	(38.8)	65	23	(35.4)	59	20	(33.9)
NIDUS intervention	203	71	(35.0)	136	44	(32.4)	102	28	(27.5)
HADS Depression Case (>=9), n(%)									
Routine care	98	28	(28.6)	65	13	(20.0)	59	13	(22.0)
NIDUS intervention	203	24	(11.8)	136	22	(16.2)	102	15	(14.7)
MCTS Total score									
Routine care	98	1.8	(2.3)	65	1.5	(2.0)	58	1.6	(2.0)
NIDUS intervention	204	1.8	(2.0)	138	1.2	(1.6)	99	1.4	(2.1)
MCTS Caseness*, n(%)									
Routine care	98	30	(30.6)	65	17	(26.2)	58	16	(27.6)
NIDUS intervention	204	64	(31.4)	138	33	(23.9)	99	19	(19.2)

* MCTS caseness defined as a score of ≥2 on any item; n = number of participants; N = total number of participants with data; SD = standard deviation

Disability Assessment for Dementia scale (DAD); brief Dimensional Apathy Scale (b-DAS); Dementia Quality of Life Scale, DEMQOL; Goal Attainment Scaling (GAS); Modified Conflict Tactics Scale (MCTS); Hospital Anxiety and Depression Scale (HADS); Neuropsychiatric inventory (NPI)

Table 4S: Sensitivity analyses adjusting for predictors of missing outcomes; ; Coefficient = Difference in means (intervention - routine care)

	Predictors of missingness	N	Coefficient	95% CI
6 months				
GAS-P	None	261	6.79	[3.48, 10.10]
GAS-C	None	261	6.63	[3.31, 9.95]
DAD score	Plwd: education	203	-4.77	[-9.69,0.16]
Demqol	Plwd: education, ethnicity	46	-2.68	[-10.85, 5.49]
Demqol proxy	Plwd: education	202	0.32	[-3.01, 3.64]
NPI	Plwd: education	205	0.68	[-3.66, 5.03]
HADS anxiety	Plwd: accommodation, education, Carer: marital status	197	-0.62	[-1.56, 0.32]
HADS depression	Plwd: accommodation, education, Carer: marital status	197	-0.44	[-1.34, 0.46]
b-DAS executive	Plwd: accommodation, education, Carer: marital status, gender	181	-0.21	[-0.71, 0.29]
b-DAS emotional	Plwd: accommodation, education, Carer: marital status	181	-0.13	[-0.70, 0.44]
12 months				
GAS-C	Plwd: age, first language	247	10.61	[6.11, 15.10]
DAD score	Plwd: living situation, education, ethnicity, Carer: marital status	160	-6.51	[-12.73, -0.29]
Demqol	Plwd: Living situation, education, marital status, gender Carer: Accommodation, relationship of carer to care recipient	26	-1.49	[-17.28,14.30]
Demqol proxy	Plwd: Living situation, education, ethnicity, Carer: Marital status	159	2.98	[-1.02,6.99]
NPI	Plwd: Living situation, education, ethnicity, age, Carer: Marital status	157	0.85	[-5.94, 7.63]
HADS anxiety	Plwd: living situation, education, ethnicity, age, Carer: Marital status	159	-0.34	[-1.42, 0.74]
HADS depression	Plwd: living situation, education, ethnicity, age, Carer: Marital status	159	0.15	[-0.74, 1.04]
BDAS executive	Plwd: Education, living situation, ethnicity, Carer: Marital status	146	-0.52	[-1.01, -0.30]
BDAS emotional	Plwd: Education, ethnicity, Carer: Marital status	146	-0.18	[-0.72, 0.35]

N = total number of participants with data; CI = Confidence Interval

Disability Assessment for Dementia scale (DAD); brief Dimensional Apathy Scale (b-DAS); Dementia Quality of Life Scale, DEMQOL; Goal Attainment Scaling (GAS); Modified Conflict Tactics Scale (MCTS); Hospital Anxiety and Depression Scale (HADS); Neuropsychiatric inventory (NPI)

Table 5S: Sensitivity analyses adjusting for predictors of missing outcomes – binary outcomes. Odds ratio compares intervention with routine care

	Predictors of missingness	N	Odds Ratio	95% CI
6 months				
BDAS initation	Plwd: accommodation, education, Carer: marital status	181	1.96	[0.72, 5.31]
MCTS	Plwd: education	200	0.93	[0.40, 2.14]
12 months				
BDAS initation	Plwd: Education, ethnicity, Carer: Marital status	146	2.15	[0.46,10.02]
MCTS	Plwd: ethnicity, education	155	0.70	[0.28, 1.74]

N = total number of participants with data; CI = Confidence Interval
brief Dimensional Apathy Scale (b-DAS); Modified Conflict Tactics Scale (MCTS);

Table 6S: Analysis of repeated measurements of secondary outcomes<br>Coefficient = Difference in means (intervention - routine care); Odds ratio compared intervention and routine care				
	**N**	**Obs**	**Coefficient**	**95% CI**
GAS-C	270	508		
6 months			6.74	[1.93,11.56]
12 months			10.42	[5.56,15.28]
DAD score	301	669		
6 months			-4.17	[-16.92,8.57]
12 months			-6.30	[-19.56,6.96]
Demqol	127	203		
6 months			-3.77	[-11.64,4.10]
12 months			0.09	[-10.40, 10.57]
Demqol proxy	302	668		
6 months			0.06	[-4.55,4.67]
12 months			4.32	[-0.62,9.26]
NPI	302	669		
6 months			-0.37	[-6.91,6.18]
12 months			0.62	[-6.41,7.64]
HADS anxiety	302	663		
6 months			-0.40	[-1.67,0.87]
12 months			-0.12	[-1.50,1.25]
HADS depression	302	663		
6 months			-0.59	[-2.25,1.07]
12 months			-0.09	[-1.82,1.64]
BDAS executive	298	635		
6 months			-0.11	[-0.57,0.35]
12 months			-0.34	[-0.83,0.15]
BDAS emotional	298	635		
6 months			-0.15	[-0.62, 0.32]
12 months			-0.16	[-0.67,0.35]
**Logistic regression**	**N**	**Obs**	**Odds Ratio**	**95% CI**
BDAS emotional	298	635		
6 months			1.02	[0.95,1.10]
12 months			0.98	[0.90,1.07]
MCTS emotional	302	662		
6 months			0.99	[0.90,1.09]
12 months			0.96	[0.86,1.06]

N = number of dyads in the model; Obs = total number of observations; please see earlier tables for abbreviations

Table 7S: (a) Adverse events

	Randomised group		
	Routine care	NIDUS-Family intervention	Total
Hospital visit (inc. A&E)	0	4	4
UTI	1	1	2
Fall	0	1	1
Paramedics called out	0	1	1
COVID-19	1	0	1
Seizures	0	1	1
Under palliative care due to cancer	0	1	1

(b) Serious Adverse Events

Number of Serious Adverse Events	Randomised group		
	Routine care	NIDUS-Family intervention	Total
Death	9	12	21
Life-threatening	0	1	1
Requires hospitalisation or prolongation of existing hospitalisation	4	21	25
Other	2	1	3

Sensitivity and supportive analyses for the primary outcome

Of the 302 dyads randomised to the study, 247 (81.7%) Plwd had the 12 month primary outcome data for analysis; 163 in the NIDUS intervention arm (79.9%) and 84 in the routine care arm (85.7%). Reasons for missingness are provided in the consort diagram.

1) To make missing at random more plausible, we refitted the primary model to obtain estimates adjusted for baseline variables (if any) associated with missingness. To identify predictors of missing data, baseline characteristics of participants with and without missing outcome data were compared using logistic regression models (with missing yes/no as the outcome). The participants first language was the only variable associated with missingness. The main analysis model has been refitted to adjust for the participants first language.

Table 1: Sensitivity analyses adjusting for predictors of missing outcome

		Randomised group	
	N	Coefficient	95% CI
Result from model adjusting for predictors of missing outcome	247	10.46	[6.08,14.84]

2) With the assumption that data are missing at random, we also carried out a sensitivity analysis using multiple imputation methods. The imputation model included baseline demographics, the six-month GAS outcome, site and facilitator. The imputations were performed by study arm. We used 18 imputations and combined the results using Rubin's rules.

Table 10: Sensitivity analyses using multiple imputation

	N	Coefficient	95% CI	p-value
Randomised group	270	10.08	[6.10,14.05]	<0.001

3) In a missing not at random sensitivity analysis, missing outcomes for those admitted to a care home or known to have died were imputed based on a worst-case scenario. For these participants the follow-up GAS performance scores for each goal were imputed with a value of -2 (representing the participants getting 'much worse' than expected) and the primary model re-fitted. We then repeated this analysis assuming a performance value of -1 for these participants. Nine plwd scores were imputed due to death. (These 9 died prior to 6 months from randomization (see consort)). There was no missing data for those who were known to have entered into a care home.

Table 11: Sensitivity analyses where missing values are considered MNAR.

	N	Coefficient	95% CI	p-value
Imputing value of -2	256	9.52	[5.33,13.71]	<0.001
Imputing value of -1	256	9.64	[5.50,13.79]	<0.001

Supportive analyses for the primary outcome

The following supportive analyses have been carried out for the primary outcome using the same modelling approaches as described previously.

1) We have performed an adjusted analysis allowing for the following pre-specified baseline factors: Recruitment time, (measured in days from the date the first participant was randomised in the study); level of functioning (measured using the total DAD score at baseline); Carer stress (measured using the total HADS score at baseline)

Table 12: Supportive analyses 1 - P-GAS at 12 months adjusted for baseline factors

	N	Coefficient	95% CI
Result from model with adjustments	247	10.40	[6.05,14.74]

2) Another supportive analysis was performed to investigate the impact of recruitment time on the treatment effect. Here the primary model was extended to include recruitment time (measured in days from the date the first participant was randomised) and an interaction between the randomisation group and recruitment time. The interaction between randomisation group and recruitment time was not statistically significant (p= 0.544). This shows that there is no evidence that the intervention performed differently depending on when the participants were recruited. The figure below shows the difference in average GAS scores between the groups for dyads recruited during three monthly intervals from recruitment of the first participant in January 2020.

Figure 2: Predictions from the model including an interaction between intervention arm and recruitment time

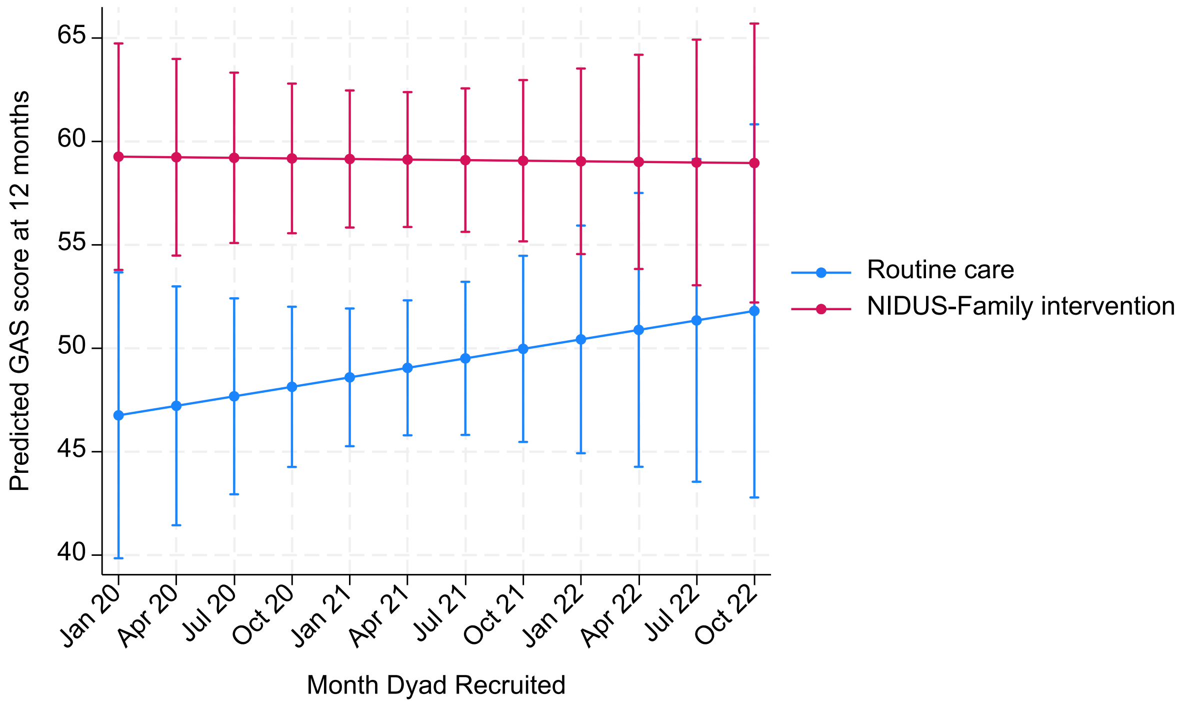

3) The baseline characteristics were examined for any concerning imbalances. The Plwd's gender and carer's gender were imbalanced. The following supportive analysis adjusts for both the Plwd and carer's gender.

Table 13: Supportive analyses 2 - P-GAS at 12 months

		Randomised group	
Adjustment	N	Coefficient	95% CI
Imbalance – Plwd and Carer gender	247	10.10	[5.60,14.59]
